# Docosahexaenoic acid complexed to human albumin in experimental stroke: neuroprotective efficacy with a wide therapeutic window

**DOI:** 10.1186/2040-7378-4-19

**Published:** 2012-09-14

**Authors:** Tiffany N Eady, Larissa Khoutorova, Kristal D Atkins, Nicolas G Bazan, Ludmila Belayev

**Affiliations:** 1 Neuroscience Center of Excellence, School of Medicine, Louisiana State University Health New Orleans, New Orleans, LA, 70112, USA; 2 Department of Neurosurgery, School of Medicine, Louisiana State University Health New Orleans, New Orleans, LA, 70112, USA; 3 Neuroscience Center of Excellence, Louisiana State University Health Sciences Center, New 2020 Gravier Street, Suite D, Orleans, LA, 70112, USA

**Keywords:** Penumbra, DHA-Alb complex, Neuroprotection, Behavior, Histopathology, Focal ischemia, Experimental stroke

## Abstract

**Background:**

Docosahexaenoic acid (DHA) complexed to human serum albumin (Alb) is neuroprotective after experimental stroke. Here we tested using lower concentrations of albumin as part of the complex to achieve neuroprotection. We found that lower Alb concentrations extend the therapeutic window of protection beyond 5 h after stroke onset.

**Methods:**

Sprague–Dawley rats were received 2 h middle cerebral artery occlusion (MCAo). The behavior was evaluated on day 1, 2, 3 and 7 after MCAo. In the dose–response study, animals were given either DHA (5mg/kg), Alb (0.63g/kg), DHA-Alb (5mg/kg + 0.32, 0.63 or 1.25 g/kg) or saline, i.v. 3 h after onset of stroke (n=6-8 per group). In the therapeutic window study, DHA-Alb (5mg/kg + 1.25g/kg) was administered i.v. at either 3, 4, 5, 6 or 7 h after onset of stroke (n=7-9 per group). Alb (1.25g/kg) was given at 3 h or 5 h and saline at 3h after onset of reperfusion. Seven days after MCAo, infarct volumes and number of GFAP, ED-1, NeuN, SMI-71 positive cells and vessels were counted.

**Results:**

Moderate DHA-Alb doses (0.63 and 1.25 g/kg) improved neurological scores compared to albumin-treated rats on days 1, 2, 3 and 7. All DHA-Alb doses (0.32, 0.63 and 1.25 g/kg) markedly reduced cortical (by 65-70%), striatal (by 52-63%) and total infarct volumes (by 60-64%) compared to native Alb group. In the therapeutic window study DHA-Alb led to improved neurological score and significant reductions of infarct volumes (especially in the cortical or penumbral region), even when treatment was initiated as late as 7 hours after onset of MCAo.

**Conclusions:**

The DHA-Alb complex affords high-grade neurobehavioral neuroprotection in focal cerebral ischemia, equaling or exceeding that afforded by native Alb or DHA, at considerably moderate doses. It has a broad therapeutic window extending to 7 h after stroke onset. Taken together, these finding support the potential clinical feasibility of administering DHA-Alb therapy to patients with acute ischemic stroke.

## Introduction

Stroke is a major cause of death and disability in industrialized countries. Pharmacological intervention of ischemic damage is critically important for controlling brain tissue deterioration. Tissue-type plasminogen activator (tPA) administered within 3 to 4.5 h of symptom onset is still the only thrombolytic agent approved for patients with ischemic stroke. However, the narrow therapeutic time window and the risk of intracerebral hemorrhage after tPA treatment pose major hurdles to its clinical use. Therefore, development of new therapeutic agents for stroke is essential.

Recent studies have revealed that omega-3 essential fatty acids (found in fish oils) may be beneficial in ameliorating cerebral ischemic injury [1,2]. Docosahexaenoic acid (DHA; 22:6, n-3) is an essential omega-3-fatty acid and is vital for proper brain function. It is also necessary for the development of the nervous system, including vision, and has potent anti-inflammatory effects [3]. Several epidemiological studies indicate that consumption of fish is associated with reduced risk of ischemic stroke and coronary heart disease [4]. Recently, we have showed that DHA treatment can protect brain tissue and promote recovery in an experimental model of acute ischemic stroke in rats [5]. In addition, DHA administration provided neurobehavioral recovery, and reduced brain infarction and edema when administered up to 5 h after focal cerebral ischemia in rats [6].

Lately, special attention has been paid to human serum albumin (Alb) therapy, which has been reported to ameliorate neuronal damage during the acute phase of stroke [7,8]. Alb has been shown to be highly neuroprotective in animal models of focal cerebral ischemia [8], global ischemia [9], hemorrhagic stroke [10,11] and traumatic brain injury [12]. With its promising neuroprotective bioactivity, Alb can markedly improve neurological function, and reduce blood brain barrier (BBB) permeability and infarct volumes when administered up to 4 h after onset of stroke. The National Institutes of Health (NIH) funded clinical trials, the ALIAS (Albumin in Acute Stroke), based on these promising studies to evaluate efficacy of Alb treatment in patients with acute ischemic stroke [13,14]. However, administration of high-dose Alb by expanding intravascular volume may lead to pulmonary edema and congestive heart failure in patients [15]. We suspected that if DHA were complexed with Alb, it might be possible to achieve neuroprotection at lower, more clinically achievable doses and extend the therapeutic window beyond 5 h after stroke onset.

## Materials and methods

### Animal preparation

The present study was conducted in accordance with the NIH guidelines for the care and use of animals in research and under protocols approved by the Institutional Animal Care and Use Committee of the Louisiana State University Health Sciences Center, New Orleans. Male Sprague–Dawley rats weighing 280–345 g (Charles River Lab., Wilmington, MA) were used in all studies. Anesthesia was induced with 3% isoflurane in a mixture of 70% nitrous oxide and 30% oxygen. All rats were orally intubated and mechanically ventilated. Body and cranial temperature were regulated to maintain temperatures at 36° to 37°C. The right femoral artery and vein were catheterized for blood sampling for arterial gases, pH, plasma glucose, and drug infusion.

### Animal model

The right middle cerebral artery (MCA) was temporarily occluded in rats with nylon filament, as we previously described [16]. In brief, the right common carotid artery (CCA) bifurcation was exposed through a midline neck incision and the occipital artery branches of the external carotid artery (ECA) were isolated, ligated and dissected. After isolation of the internal carotid artery (ICA), a 3–0 monofilament coated with poly-L-lysine was advanced through the ICA to the MCA until mild resistance was felt. The neck incision was closed with a silk suture and the animals were then allowed to recover. After 2 h of MCA occlusion (MCAo), rats were reanesthetized with the same anesthetic combination and intraluminal sutures were carefully removed. Animals survived for 7 days with free access to food and water.

### Behavioral testing

Behavioral tests were performed by an observer blinded to the treatment groups at 60 minutes during MCAo and then on days 1, 2, 3 and 7 after MCAo. The battery consisted of two tests that have been used previously [16] to evaluate various aspects of neurologic function: 1) the postural reflex test, to examine upper body posture while the animal is suspended by the tail, and 2) the forelimb placing test, to examine sensorimotor integration in forelimb placing responses to visual, tactile and proprioceptive stimuli. Neurological function was graded on a scale of 0 (normal) to 12 (maximal injury) as previously described [16]. Only animals with a high-grade neurological deficit (10 or greater) were used in this study.

### Treatment and experimental groups

Docosahexaenoic acid (DHA) in acid form (Cayman Chemical, Ann Arbor, MI) was physically complexed to human albumin by incubating 20 ml of human serum albumin (25%; Baxter, Westlake Village, CA) with 5 mg DHA/g albumin (molar ratio =0.2) as previously described [17].

### Dose–response study

Animals were randomly assigned to six treatment groups: DHA (5mg/kg), human serum albumin (Alb; 25%, 0.63g/kg), DHA-Alb (5mg/kg + 0.32 g/kg, 5mg/kg + 0.63 g/kg or 5mg/kg + 1.25 g/kg), or sodium chloride (0.9%, 5ml/kg). The respective agent was administered into femoral vein at a constant rate over 3 minutes by infusion pump at 3 h after onset of stroke (n=6-8 per group).

### Therapeutic window study

In the therapeutic window study, DHA-Alb (5mg/kg +1.25 g/kg) was administered into femoral vein at either 3, 4, 5, 6 or 7 h after onset of stroke (n=7-9 per group). Vehicle group received Alb (25%, 1.25g/kg) at 3 or 5 h after onset of reperfusion. The control group received sodium chloride (0.9%, 5ml/kg) at 3 h after onset of stroke (n=6).

### Histopathology and immunostaining

Animals were perfused with 4% paraformaldehyde on day 7, and brains were removed and embedded in a gelatin matrix using MultiBrain™ Technology (NeuroScience Associates, Knoxville, TN) as previously described [18]. Coronal sections were stained with thionine (Nissl), digitized at nine standardized coronal levels, and the area of infarction was measured and analyzed using MCID™ Core imaging software (Linton, Cambridge, United Kingdom) as previously described [16]. An investigator blinded to the experimental groups then outlined the zones of infarction (which were clearly demarcated) as well as the left and right hemispheres of each section. Infarct volume was calculated as the integrated product of cross-sectional area and inter-section distance and corrected for brain swelling.

Immunohistochemical procedures were performed on the adjacent sections to identify specific vascular and neuronal elements in the ischemic core and penumbra. The following antibodies were used: rat BBB (SMI-71, Sternberger Monoclonals, Inc., Baltimore, MD) as a vascular marker, glial fibrillary acid protein (GFAP, Santa Cruz, SDS Biosciences, Sweden) to label reactive astrocytes, and Cd68/ED-1 (Serotec, Raleigh, NC) to activate microglia/macrophages. Numbers of GFAP, ED1 and SMI-71 immunopositive vessels were counted in the cortex and subcortex at the level of the central lesion (bregma level −0.3 mm). Data were expressed as numbers of positive cells and vessels per high-power microscopic field (magnification X 40).

### Statistical analysis

Data are presented as mean values ± SEM. Repeated measure analysis of variance (ANOVA) followed by Bonferroni procedures were used for multiple comparisons. Two-tailed Student’s *t* tests were used for two-group comparisons. Differences at *P*<0.05 were considered statistically significant.

## Results

### Physiological variables

Rectal and cranial (temporalis muscle) temperatures, arterial blood gases, and plasma glucose showed no significant differences between animals. Albumin (1.25 g/kg) and DHA-Alb therapy (5mg/kg + 1.25 g/kg) led to the expected moderate reduction in hematocrit compared to the vehicle group (Table 1). There were no adverse behavioral side effects observed after DHA, Alb or DHA-Alb administration in all groups.

**Table 1:** Physiological variables

	**Dose-response study**						**Therapeutic window study**									
	**Saline**	**DHA 5 mg/kg**	**Alb 0.63 g/kg**	**DHA-Alb**			**Saline**	**DHA 5 mg/kg**	**Alb, 1.25 g/kg**	**DHA-Alb**			**Alb, 1.25g/kg**	**DHA-Alb**		
				**0.32 g/kg**	**0.63 g/kg**	**1.25 g/kg**			**3h**	**2h**	**3h**	**4h**	**5h**	**5h**	**6h**	**7h**
	**(n=6)**	**(n=7)**	**(n=6)**	**(n=7)**	**(n=5)**	**(n=7)**	**(n=7)**	**(n=8)**	**(n=8)**	**(n=8)**	**(n=8)**	**(n=7)**	**(n=9)**	**(n=7)**	**(n=9)**	**(n=8)**
**Before MCAo (15 min)**																
Rectal temperature (^o^C)	36.6 Â± 0.1	37.3 Â± 0.1	37.1 Â± 0.1	36.9 Â± 0.1	37.3 Â± 0.2	37.1 Â± 0.1	36.8 Â± 0.1	36.9 Â± 0.1	37.2 Â± 0.1	37.0 Â± 0.1	36.9 Â± 0.1	36.9 Â±0.1	37.0 Â± 0.1	36.8 Â± 0.1	37.0 Â± 0.1	36.8 Â± 0.1
Cranial temperature (^o^C)	36.6 Â± 0.1	36.8 Â± 0.1	36.9 Â± 0.2	36.3 Â± 0.1	36.8 Â± 0.2	36.6 Â± 0.2	36.7 Â± 0.3	36.8 Â± 0.2	37.1 Â± 0.1	36.9 Â± 0.1	36.6 Â± 0.2	36.8 Â± 0.1	36.5 Â± 0.2	36.5 Â± 0.3	36.8 Â± 0.1	36.7 Â± 0.2
pH	7.4 Â± 0.03	7.4 Â± 0.01	7.4 Â± 0.01	7.4 Â± 0.01	7.4 Â± 0.01	7.4 Â± 0.01	7.5 Â± 0.01	7.4 Â± 0.01	7.5 Â±0.01	7.5 Â± 0.00	7.5 Â± 0.01	7.4 Â± 0.01	7.4 Â± 0.01	7.4 Â± 0.01	7.5 Â± 0.01	7.4 Â± 0.01
PO2, mm Hg	120 Â± 7	106 Â± 7	115 Â± 9	115 Â± 7	113 Â± 8	105 Â± 5	102 Â± 9	109 Â± 6	104 Â± 6	122 Â± 10	96 Â± 5	106 Â± 7	102 Â± 7	103 Â± 6	108 Â± 14	107 Â± 10
PCO2, mm Hg	40.0 Â± 2.0	39.0 Â± 1.0	38.8 Â± 1.0	38.3 Â± 0.7	39.4 Â± 0.9	40.7 Â± 0.5	37.5 Â± 0.9	38.9 Â± 0.7	37.6 Â± 1.0	38.0 Â± 0.9	39.0 Â± 0.7	38.7 Â± 0.8	38.6 Â± 0.7	39.6 Â± 1.6	47.3 Â± 8.4	39.4 Â± 1.0
Plasma glucose, mg/dL	151 Â± 14	154 Â± 8	175 Â± 13	181 Â± 14	183 Â± 18	193 Â± 15	173 Â± 13	132 Â± 1.0	162 Â± 14	161 Â± 8	160 Â± 7	147 Â± 7	171 Â± 10	156 Â± 11	168 Â± 11	156 Â± 8
Hematocrit, %	44 Â± 1.0	43 Â± 0.3	44 Â± 0.9	44 Â± 0.8	44 Â± 0.8	44 Â± 0.5	42 Â± 1.0	45 Â± 1.2	45 Â± 1.2	43 Â± 0.4	45 Â± 1.2	43 Â± 1.0	46 Â± 0.7	44 Â± 0.8	45 Â± 12	43 Â± 1.7
Body weight (g)	334 Â± 5	324 Â± 8	304 Â± 11	320 Â± 7	309 Â± 6	304 Â± 7	334 Â± 14	302 Â± 4	327 Â± 6	317 Â± 9	306 Â± 8	333 Â± 7	335 Â± 13	321 Â± 11	326 Â± 7	330 Â± 11
**During MCAo (15 min)**																
Rectal temperature (^o^C)	36.8 Â± 0.1	37.2 Â± 0.1	37.0 Â± 0.1	37.0 Â± 0.1	37.3 Â± 0.1	37.1 Â± 0.1	37.2 Â±0.1	36.9Â±0.1	37.0Â±0.1	37.2Â±0.1	37.3Â±0.1	37.2Â±0.1	37.1Â±0.1	37.0Â±0.2	37.3Â±0.1	37.1Â±0.1
Cranial temperature (^o^C)	36.6 Â± 0.1	36.3 Â± 0.1	36.6 Â± 0.1	36.6 Â± 0.1	36.7 Â± 0.2	36.6 Â± 0.1	37.0 03	37.0Â±0.0	37.6Â±0.5	37.0Â±0.0	37.0Â±0.2	36.9Â±0.1	36.9Â±0.1	36.9Â±0.1	36.6Â±0.2	37Â±0.0
pH	7.4 Â± 0.01	7.4 Â± 0.01	7.4 Â± 0.01	7.4 Â± 0.01	7.4 Â± 0.02	7.5 Â± 0.01	7.4 Â± 0.1	7.4Â±0.01	7.4Â±0.01	7.5Â±0.02	7.4Â±0.02	7.4Â±0.02	7.4Â±0.00	7.4Â±0.01	7.4Â±0.01	7.4Â±0.02
PO2, mm Hg	112Â±8	97 Â± 5	99 Â± 7	104 Â± 5	108 Â±7	99 Â± 4	98 Â± 1	98Â±4	103Â±5	105Â±8	96Â±5	107Â±9	113Â±9	101Â±6	102Â±6	97Â±6
PCO2, mm Hg	40.0 Â± 2.0	40.0 Â± 0.0	39.2 Â± 0.8	40.7 Â± 1.0	39.0 Â± 0.9	38.4 Â± 0.8	41.0 Â± 1.4	39.2Â±0.6	39.1Â±0.7	39.8Â±1.0	40.0Â±1.3	39.5Â±0.8	39.7Â±0.6	39.8Â±0.6	39.3Â±0.7	40.3Â±1.7
Plasma glucose, mg/dL	141 Â± 7	159 Â± 7	176 Â± 13	181 Â± 14	202 Â± 19	189 Â± 9	160 Â± 10	148Â±15	150Â±11	156Â±9	145Â±27	151Â±9	169Â±11	159Â±10	149Â±8	147Â±8
Hematocrit, %	44 Â± 1.0	43 Â± 0.4	44 Â± 1.0	44 Â± 0.7	43 Â± 0.4	44 Â± 0.5	43 Â± 1.4	46Â±0.5	44.6Â±1.4	45Â±0.8	46Â±0.9	44Â±1.0	46Â±0.9	44Â±1.1	47Â±1.1	46Â±1.3
**15 min after treatment**																
Rectal temperature(^o^C)	37.2Â±0.3	36.6Â±0.2	37.2Â±0.1	37.2Â±0.3	37.1Â±0.1	37.0Â±0.1	36.7Â±0.1	37.0Â±0.1	37.2Â±0.2	36.7Â±0.1	37.0Â±0.2	37.1Â±0.1	37.1Â±0.2	37.1Â±0.2	37.1Â±0.2	37.2Â±0.1
Cranial temperature(^o^C)	37.2Â±0.2	36.6Â±0.2	37.5Â±0.2	37.9Â±0.4	37.7Â±0.3	37.2Â±0.2	36.1Â±0.1	36.1Â±0.1	36.8Â±0.3	36.3Â±0.2	36.4Â±0.3	36.3Â±0.2	36.8Â±0.2	36.3Â±0.3	36.1Â±0.1	36.8Â±0.2
Hematocrit, %	45Â±1.1	44Â±0.9	37Â±0.3	40Â±0.6	37Â±0.5	35Â±0.7*	43Â±1.4	44Â±1.0	35Â±1.4*	35Â±0.8*	38Â±0.6*	35Â±0.6*	36Â±0.8*	36Â±0.8*	36Â±0.6*	36Â±1.1*
**1 day after treatment**																
Rectal temperature (^o^C)	38.3Â±0.2	38.3Â±0.2	38.4Â±0.1	37.8Â±0.2	38.0Â±0.3	37.7Â±0.1	38.0Â±0.2	38.0Â±0.2	38.1Â±0.3	37.8Â±0.5	37.0Â±0.2	37.7Â±0.3	38.0Â±0.3	37.6Â±0.2	37.8Â±0.1	37.7Â±0.2
Body weight(g)	301Â±6	300Â±7	270Â±10	294Â±7	287Â±7	281Â±6	299Â±14	275Â±7	304Â±9	303Â±10	283Â±10	304Â±10	303Â±15	294Â±16	308Â±9	303Â±14
**7 days after treatment**																
Rectal temperature (^o^C)	37.8Â±0.3	38.0Â±0.2	35.1Â±1.4	37.6Â±0.3	37.8Â±0.2	37.8Â±0.1	37.7Â±0.1	37.5Â±0.2	37.7Â±0.2	37.7Â±0.1	36.7Â±0.9	37.4Â±0.5	35.8Â±1.3	37.2Â±0.2	37.4Â±0.1	36.4Â±1.2
Hematocrit, %	44Â±1.3	40Â±0.8	47Â±0.2	41Â±0.7	45Â±0.5	43Â±0.8	45Â±1.2	41Â±1.1	40Â±1.1	42Â±0.9	39Â±0.5	39Â±0.3	41Â±0.8	39Â±0.8	40Â±0.7	39Â±1.0
Body weight (g)	305Â±16	319Â±17	219Â±12	305Â±19	307Â±12	310Â±7	318Â±18	297Â±16	331Â±14	294Â±25	311Â±20	307Â±21	333Â±24	304Â±24	345Â±7	321Â±21

Values are mean Â± SEM.

MCAo, middle cerebral artery occlusion.

*different from saline group (p<0.05, Student’s t-test).

### Dose response study

**Figure 1:**
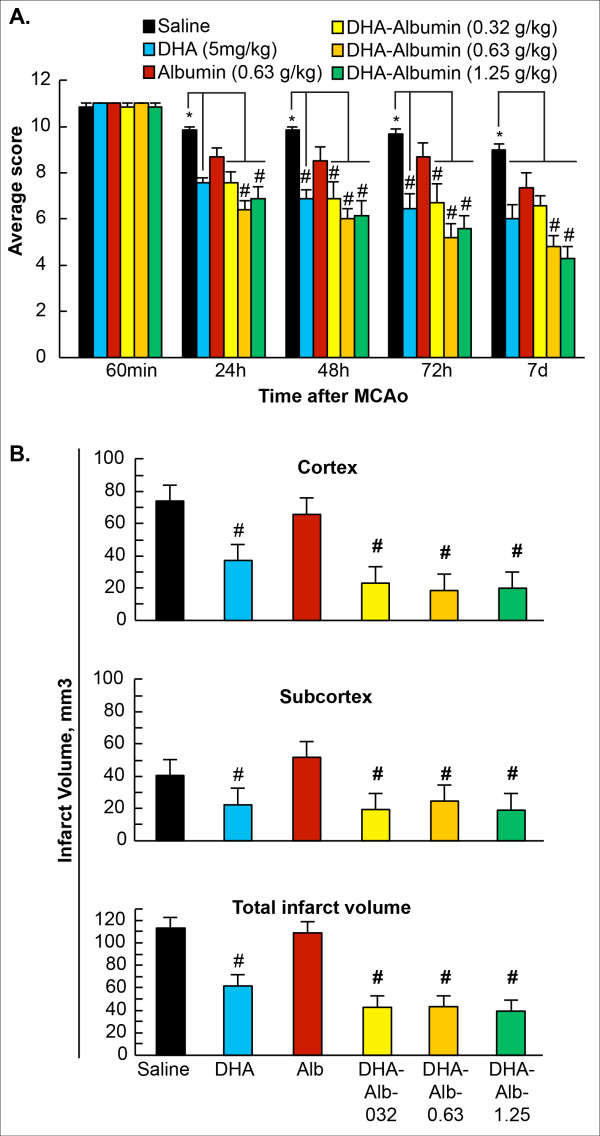
**Dose response study: (A) Total neurological score (normal score=0, maximal score=12) during MCAo and at various times after treatment.** Saline, DHA 5 mg/kg), Albumin (0.63 g/kg) or DHA-Albumin (5 mg/kg + 0.32, 0.63 or 1.25 g/kg) treatment was administered at 3 h after onset of ischemia. (**B**) Histopathology on day 7 of survival. All doses of DHA-Alb significantly reduced cortical, subcortical and total (cortical and subcortical) corrected infarct volume compared to Alb-treated rats. Data are mean ±SEM; n=5-7 per group. *P <0.05 versus saline group; ^#^*P* <0.05 versus Alb group (repeated-measures ANOVA followed by Bonferroni tests).

**Figure 2:**
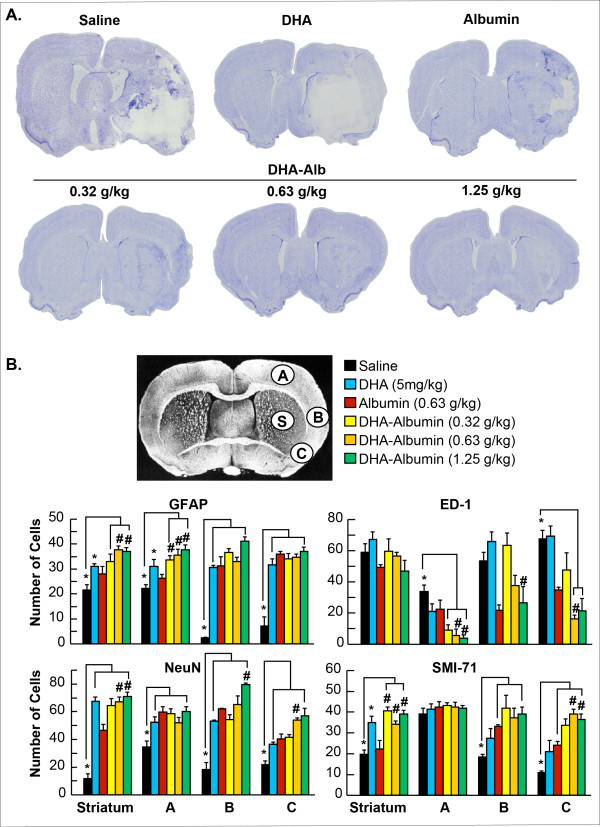
**Histopathology: (A) Computer-generated MosaiX processed images (Zeiss Axio Imager.M1; AxioVision Release 4.6.3) of Nissl stained paraffin-embedded brain sections from rats treated with saline, DHA (5 mg/kg), Albumin (0.63 g/kg) or DHA-Albumin (5 mg/kg + 0.32, 0.63 or 1.25 g/kg).** Saline and Alb-treated rats show large cortical and subcortical infarction. Rats treated with DHA shows less extensive damage. In contrast, DHA-Albumin treated rats show very small infarction, mostly in the subcortical area. (**B**) Coronal brain diagram showing locations of regions for cell counts in cortex (A, B and C) and subcortex (S). Number of GFAP positive astrocytes, ED-1 positive microglia cells, NeuN positive neurons and SMI-71 positive vessels were counted on day 7 after 2 h of MCAo. DHA-Alb treatment decreased ED-1, increased NeuN, GFAP positive cell counts and SMI-71 positive vessels. Data are mean ±SEM; n=5-7 per group. *P <0.05 versus saline group; ^#^*P* <0.05 versus Alb group (repeated-measures ANOVA followed by Bonferroni tests).

Animals treated with moderate doses of DHA-Alb (0.63 and 1.25g/kg) significantly improved the neurological score compared to Alb-treated rats on days 1, 2, 3 and 7 (Figure 1A). The lowest dose of DHA-Alb (0.32g/kg) was not different from the Alb-treated group on day 7. In addition, DHA treatment improved behavioral score compared to the Alb-treated rats, but only on day 2 (by 19%) and day 3 (by 26%) (Figure 1A). All doses of DHA-Alb significantly reduced cortical and subcortical infarct volumes in all treated groups compared to Alb-treated rats (Figure 1B). In addition, all doses of DHA-Alb also reduced total corrected infarct and also cortical and subcortical infarct volumes as well (Figure 1B). DHA treatment alone reduced infarct volumes compared to the Alb-treated group, but less significantly than DHA-Alb complex. Representative images of Nissl stained brain sections from all groups are presented in Figure 2A. The brains of saline and albumin-treated animals exhibited a consistent pannecrotic lesion involving both cortical and subcortical regions of the right hemisphere, characterized microscopically by the destruction of neuronal, glial, and vascular elements. By contrast, infarct size was dramatically reduced by DHA-Alb therapy (0.63 and 1.25g/kg) (Figure 2A). DHA and the lowest dose of DHA-Alb (0.32g/kg) also reduced infarct volume, but at a lesser rate compared to rats treated with moderate doses of DHA-Alb. DHA-Alb treatment decreased ED-1 positive microglia cells in the penumbra, and increased NeuN positive neurons, GFAP positive astrocytes and SMI-71 positive vessels in the ischemic penumbra and core (subcortex) (Figure 2B).

### Therapeutic window study

**Figure 3:**
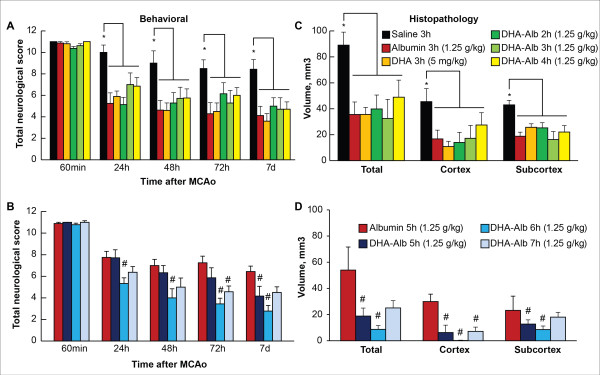
**Therapeutic window study.** Saline and DHA (5 mg/kg) were administered at 3h, Albumin (1.25 g/kg) at 3 h or 5 h and DHA-Albumin (5 mg/kg + 1.25 g/kg) at 2, 3, 4, 5, 6 or 7 h after onset of MCAo. (**A**) Total neurological score in Alb, DHA and DHA-Alb (at 2, 3 and 4h) and (**B**) Alb, DHA-Alb (at 5, 6 and 7 h) treated groups. Total, cortical and subcortical infarct volumes in (**C**) Alb, DHA and DHA-Alb (at 2, 3 and 4 h) and (**D**) Alb, DHA-Alb (at 5, 6 and 7 h) treated groups. DHA-Alb therapy led to significantly improved neurological score and highly significant reductions of total, cortical and subcortical infarct volumes, even when treatment was initiated as late as 7 h after onset of MCAo. Data are mean ±SEM; n=7-9 per group. *P <0.05 versus saline group; ^#^*P* <0.05 versus Alb group (repeated-measures ANOVA followed by Bonferroni tests).

Alb and DHA-Alb treatment started at 2, 3 and 4 h after onset of stroke significantly improved neurological scores compared to saline, but there was no significant difference between DHA-Alb and Alb groups (Figure 3A). In rats given DHA-Alb at 5, 6 or 7 h after stroke, neurobehavioral improvement exceeded that of native Alb throughout the 7-day survival period (Figure 3B). Infarct volumes were significantly reduced by Alb, DHA and DHA-Alb treatment (administered at 2, 3 and 4 h) compared to saline treated group (Figure 3C), but there was no significant difference between DHA-Alb and Alb groups. When DHA-Alb treatment was initiated as late at 5, 6 or 7 h after onset of stroke, infarct volumes, especially in the cortical area, were dramatically reduced compared to Alb treated group even when treatment was delayed until 7 h (Figure 3D).

## Discussion

In the first part of this study we have shown that DHA-Alb therapy substantially improves behavioral function, markedly reduces the volume of cerebral infarction, and promotes cell survival in animals with acute ischemic stroke when administered promptly at moderate doses, which are potentially amenable to clinical applications. In the second part of the study we have demonstrated the existence of a broad therapeutic window of neuroprotective efficacy with moderate-dose DHA-Alb therapy, such that treatment initiated even 7 hours after stroke onset of ischemia is highly effective.

Cerebral ischemia-reperfusion results in the rapid accumulation of free fatty acids (FFA), including arachidonic acid (AA; 20:4 n-6) and DHA, which are released from membrane phospholipids. Both AA and DHA are polyunsaturated fatty acids highly enriched in phospholipids from synaptic membranes, where they are reservoirs of potent signaling molecules and essential for overall membrane function [1]. DHA is necessary for ion channels, receptors, and transporters to maintain their proper physical conformation and is involved in memory, synaptic membrane biogenesis and function, and neuroprotection [5,19,20]. DHA is the precursor of neuroprotectin D1 (NPD1), which has been associated with reduction of neuroinflammation and activation of antiapoptotic pathways, two main mechanisms of action implicated in ischemic stroke [5,21,22]. Our studies reveal an early increase in unesterified (free) DHA in brain ischemia [19], which initiates a pathway for docosanoid biosynthesis [21]. Since inflammation is at the root of many chronic diseases, DHA treatment has been demonstrated to have beneficial effects in patients with coronary heart disease, rheumatoid arthritis, osteoporosis, sepsis, asthma, cancer and age-related macular degeneration, but its potential benefit in stroke was not known [23]. Recently, we have shown that DHA therapy in low (3.5 or 7 mg/kg) and medium (16 or 35 mg/kg) doses improves neurological and histological outcomes following focal cerebral ischemia [5]. Thus, we selected a dose in the middle of the effective dose range (5 mg/kg) for our studies. In addition, we demonstrated that DHA or saline administered intravenously at 3, 4 and 5 h after the onset of stroke reduced cerebral infarction and brain swelling, and facilitated neurobehavioral recovery even when treatment was delayed by up to 5 h [6].

Human serum albumin has been used for many years in the management of a diverse range of medical and surgical issues [24]. Alb administration has multiple effects including volume expansion, an increase in colloid osmotic pressure, and hemodilution. It is used in multiple clinical settings such as hypovolemia, shock, burns, surgery, trauma, cardiopulmonary bypass, acute respiratory distress syndrome, hemodialysis, acute liver failure, and ascites [25].

Preclinical studies in rodent models of ischemic stroke have established that that administration of Alb at high doses (25%, 2.5 g/kg, i.v.) decreases infarct volume, reduces brain swelling [7,26] and improves local cerebral perfusion in affected tissue [27] Moderate doses of Alb therapy (0.63 or 1.25 g/kg) also improve neurological function and reduce infarct volume and brain swelling, even when treatment is delayed up to 4 hours after onset of ischemia in rats [8]. Albumin produces its neuroprotective effect through several mechanisms including brain swelling amelioration [26], BBB permeability reduction [10], local vascular dynamic and tissue perfusion improvement [28,29] antioxidant activity [30], antithrombotic effects and anti-inflammatory activity [31], inhibiting endothelial apoptosis and maintenance of normal astrocytic function [32] and providing essential fatty acids to the injured brain [20].

Alb is currently under clinical trials for treatment of focal cerebral ischemia [13,14] and subarachnoid hemorrhage [33]. Phase I and II clinical trials have reported that higher doses of Alb correlated with better outcomes in subjects with acute ischemic stroke [15,34], and a Phase III trial has begun [13,14]. The only Alb-related adverse event encountered was mild-to-moderate pulmonary edema, which occurred in approximately 13% of patients [15]. We suspected that if DHA were complexed with Alb, it might be possible to achieve neuroprotection at lower and therefore more clinically achievable, doses and extend the therapeutic window beyond 5 h after stroke onset. The results of our experiments show that animals treated with moderate doses of DHA-Alb (0.63 and 1.25g/kg) significantly improved the neurological score compared to native Alb by 21 and 26% on day 1; by 28 and 29% on day 2; by 36 and 40% on day 3; and by 42 and 35% on day 7, respectively. All DHA-Alb doses markedly reduced infarct volumes compared to native Alb group. In the therapeutic window study, even when treatment was initiated as late as 7 h after onset of MCAo, DHA-Alb therapy led to significantly improved neurological scores and significant reductions of infarct volumes. Due to the challenges of treating patients in the timely manner, an extension of the therapeutic window to 7 h will offer great opportunities to clinicians treating ischemic stroke victims.

Acute stroke causes an irreversibly damaged ischemic core, and salvageable surrounding tissue called the ischemic penumbra [35]. The ischemic core is characterized by severe reduction of local cerebral blood flow (below 20%), disruption of ion homeostasis, massive release of glutamate, and activation of proteases and endonucleases, which may contribute to the acute neuronal death observed in the ischemic hemisphere [36]. The penumbra of ischemic stroke refers to the regions of brain tissue, surrounding the ischemic core, where blood flow is sufficiently reduced (below 40%) to result in hypoxia severe enough to arrest physiological function, but not so complete as to cause irreversible failure of energy metabolism and cellular necrosis [37]. The penumbra, which accounts for almost one-half of the initial ischemic lesion [38], deteriorates over a few hours following the initial ischemic core damage and is therefore the target of therapeutic intervention [36,39]. Recent studies showed that DHA treatment decreased ED-1 –positive microglia/macrophages, increased GFAP –positive reactive astrocytes and NeuN positive neurons in the ischemic penumbra and core [11]. Our study demonstrates that DHA-Alb complex was able to salvage the ischemic penumbra. The effect of DHA-Alb was evident in the cortical (penumbral region) of the infarct through a significant 79% reduction of cortical infarct volume when administered at 5 h, 98% reduction at 6 h, and 76% at 7 h, as compared to Alb treated rats. This effect was associated with attenuated cellular death of both astrocytes and neurons and fewer activated microglia, as compared to vehicle-treated rats. GFAP expression was found in the boundary zone of infarct and was localized to the same areas where neurons are destined to survive the ischemic insult (as detected by NeuN positive cells count). These results indicate that DHA-Alb protects not only neurons but also astrocytes, which are critical for the maintenance and protections of neurons via secretion of growth factors and other neurotrophic mediators.

We have shown here that all DHA-Alb doses used in this study were highly neuroprotective, the therapeutic window was extended to 7 h by complexing DHA to human Alb (1.25 g/kg), and delayed DHA-Alb treatment (at 5-7h) was more effective than early treatment (at 2-4h) compared to native Alb. The exact mechanisms of DHA-Alb neuroprotection remain obscure. We can speculate however, that complexing DHA to human Alb provides additive neuroprotection in experimental stroke. Lipidomic analysis of DHA-Alb treated postischemic brains revealed a large accumulation of NPD1 in the ipsilateral hemisphere at 20 h of reperfusion after 2 h of MCAo in rats [17]. NPD1 is a potent inhibitor of polymorphonuclear leukocyte infiltration and pro-inflammatory gene expression, and an overall mediator of neuroprotection [6,21]. In contrast NPD1 levels did not change in either ipsilateral or contralateral hemispheres in Alb treated rats [17]. Studies shows that DHA-Alb can also prevent injury to cultured human retinal pigment epithelial cells triggered by oxidative stress, and that this effect is mediated by NPD1 [40]. Albumin is actively involved in plasma transport of FFA [41]. Because transient MCAo triggers a massive loss of phospholipid-acyl groups, the systemic supply of FFA to the brain, mainly AA and DHA, may be essential to support the repair of neuronal membranes [42]. The direct delivery of FFA to brain cells by Alb may be favored by ischemia-induced disruption of the BBB [43]. In fact, previous studies have reported that the integrity of the BBB is preserved at 3–4 h after MCAo [44] but that progressive acute disruption of the BBB occurs between 5–6 h following MCAo [43]. In addition, immunohistochemical analysis reveals that following MCAo, viable-appearing cortical neurons and endothelial cells within the ischemic zone take up Alb [45]. Thus, this may be evidence in favor of a direct protective role of Alb, not only as a free-radical and toxic-product scavenger [31], but also as a supplier of DHA.

## Conclusion

The current study has shown that moderate doses of DHA-Alb complex affords high-grade neuroprotection in focal cerebral ischemia, equaling or exceeding that afforded by native Alb or DHA. Importantly DHA-Alb therapy initiated as late as 7 h after onset of stroke improved behavioral scores and reduced infarct volume. This 7-hour time frame is clinically relevant in that it is logistically difficult to institute therapy in many patients with acute stroke at earlier times. We therefore suggest that this agent offers great promise in the therapy of cerebral ischemia and we propose that it may now be appropriate to consider early-phase clinical trials in patients with acute ischemic stroke.

## Competing interests

The authors declare no conflicts of interest.

## Authors’ contributions

All authors read and approved the final manuscript.
